# Supplementary material for: Long-read sequencing for 29 immune cell subsets reveals disease-linked isoforms
Source: Nat Commun. 2024 May 28;15:4285. doi: 10.1038/s41467-024-48615-4 (PMC11133395; doi:10.1038/s41467-024-48615-4)
Supplement: Supplementary file 10 — Reporting Summary [file 41467_2024_48615_MOESM10_ESM.pdf]

nature portfolio

Corresponding author(s): Yuta Kochi

Last updated by author(s): Apr 10, 2024

Reporting Summary

Nature Portfolio wishes to improve the reproducibility of the work that we publish. This form provides structure for consistency and transparency in reporting. For further information on Nature Portfolio policies, see our Editorial Policies and the Editorial Policy Checklist.

Statistics

For all statistical analyses, confirm that the following items are present in the figure legend, table legend, main text, or Methods section.

n/a	Confirmed	
☐	☒	The exact sample size (*n*) for each experimental group/condition, given as a discrete number and unit of measurement
☐	☒	A statement on whether measurements were taken from distinct samples or whether the same sample was measured repeatedly
☐	☒	The statistical test(s) used AND whether they are one- or two-sided<br>*Only common tests should be described solely by name; describe more complex techniques in the Methods section.*
☐	☒	A description of all covariates tested
☐	☒	A description of any assumptions or corrections, such as tests of normality and adjustment for multiple comparisons
☐	☒	A full description of the statistical parameters including central tendency (e.g. means) or other basic estimates (e.g. regression coefficient) AND variation (e.g. standard deviation) or associated estimates of uncertainty (e.g. confidence intervals)
☐	☒	For null hypothesis testing, the test statistic (e.g. *F*, *t*, *r*) with confidence intervals, effect sizes, degrees of freedom and *P* value noted<br>*Give P values as exact values whenever suitable.*
☒	☐	For Bayesian analysis, information on the choice of priors and Markov chain Monte Carlo settings
☒	☐	For hierarchical and complex designs, identification of the appropriate level for tests and full reporting of outcomes
☐	☒	Estimates of effect sizes (e.g. Cohen's *d*, Pearson's *r*), indicating how they were calculated

Our web collection on statistics for biologists contains articles on many of the points above.

Software and code

Policy information about availability of computer code

Data collection	No software was used for data collection.
Data analysis	We used publicly available software for the data analysis (Guppy (v5.0.15), minimap2 (v2.17), PLINK (v1.9 or v.2.0 depending on the analysis), SAMtools (v1.9), Flair (v1.5), SQANTI3 (v4.2), SHAPEIT (v2.r904), MiniMac3 (v2.0.1), StringTie2 (v2.1.6), kallisto (v0.46.1), RBPmap (v1.2), RepeatMasker (v4.1.2), ViennaRNA Package (v2.5.0), LDSC (v1.0.1), VSEARCH (v2.21.1), LeafCutter (v0.2.7), DaPars2 (v2.1), R (v3.6), peer (v1.0), IsoformSwitchAnalyzeR (v1.18.0), MatrixEQTL (v2.1), and coloc (v3.2.1)). Source code is available at GitHub (https://github.com/juninamo/TRAILS). Default parameters were used unless otherwise stated in the Method section.

For manuscripts utilizing custom algorithms or software that are central to the research but not yet described in published literature, software must be made available to editors and reviewers. We strongly encourage code deposition in a community repository (e.g. GitHub). See the Nature Portfolio guidelines for submitting code & software for further information.

Data

Policy information about availability of data

All manuscripts must include a data availability statement. This statement should provide the following information, where applicable:

- Accession codes, unique identifiers, or web links for publicly available datasets
- A description of any restrictions on data availability
- For clinical datasets or third party data, please ensure that the statement adheres to our policy

Isoform expression data have been deposited in the DNA Data Bank of Japan (DDBJ) via the National Bioscience Database Center (NBDC) Human Database under accession code DRA016285 (https://ddbj.nig.ac.jp/resource/sra-submission/DRA016285). The MS raw data and analysis files have been deposited in the ProteomeXchange Consortium via the jPOST partner repository with the data set identifier PXD040962 (https://proteomecentral.proteomexchange.org/cgi/GetDataset?ID=PXD040962).

Research involving human participants, their data, or biological material

Policy information about studies with human participants or human data. See also policy information about sex, gender (identity/presentation), and sexual orientation and race, ethnicity and racism.

Reporting on sex and gender	We sorted peripheral blood mononuclear cells into 29 immune cell subsets from a healthy volunteer (42-year-old female). As a validation data and examine the sex difference, we sequenced peripheral blood mononuclear cells from a healthy volunteer (40-year-old male).
Reporting on race, ethnicity, or other socially relevant groupings	The selection of volunteers, a 42-year-old female and a 40-year-old male, was based on health status rather than any socially constructed or socially relevant categorizations. In our study, age and sex were not used as proxies for any other variable, such as socioeconomic status, but were recorded as biological variables relevant to the immune cell analysis. Confounders such as potential batch effects from cell sorting or RNA extraction were controlled for through standardized protocols and equipment across all samples, ensuring consistency. Moreover, we applied rigorous quality control measures at each step of the process, which are outlined in the Methods section of our manuscript.
Population characteristics	PBMC samples for long-read sequencing are obtained from Japanese people.
Recruitment	We acknowledge the potential for self-selection bias in our study, as it relied on volunteers. Individuals who choose to participate in a study might differ from those who do not in ways that could affect the results, such as their health awareness, access to medical care, or interest in medical research. In our case, participants were healthy volunteers, which might not be representative of the general population's immune cell profiles. Therefore, the findings might not be generalizable to individuals with underlying health conditions or different demographic backgrounds.
Ethics oversight	This study was approved by the Ethics Committees of the Medical Research Institute, Tokyo Medical and Dental University and RIKEN Center for Integrative Medical Sciences. Written informed consent was obtained from each volunteer.

Note that full information on the approval of the study protocol must also be provided in the manuscript.

Field-specific reporting

Please select the one below that is the best fit for your research. If you are not sure, read the appropriate sections before making your selection.

☒ Life sciences ☐ Behavioural & social sciences ☐ Ecological, evolutionary & environmental sciences

For a reference copy of the document with all sections, see nature.com/documents/nr-reporting-summary-flat.pdf

Life sciences study design

All studies must disclose on these points even when the disclosure is negative.

Sample size	The scope of our datasets and the methodological choices made were strategically aligned with our study's objectives and the current state of technological and financial feasibility. This approach allowed us to explore and validate the complex landscape of immune cell splicing, acknowledging the limitations posed by inter-individual variability as a direction for future research.
Data exclusions	No data was excluded.
Replication	We additionally collected PBMC from a 40-year-old male and applied long-read RNA sequencing using the latest ONT platform (PromethION R10.4.1, V14 chemistry). This dataset allowed us to compare the isoforms in PBMC between female and male. Meanwhile, it also enabled us to validate the presence of isoforms in our data, owing to its higher quality of sequences, higher read depth, and comprehensive coverage of all the immune subsets by PBMC.
Randomization	Randomization of the experimental design does not apply for our analysis; The nature of our study, focusing on the comprehensive analysis of immune cell transcriptomes and the diversity of alternative splicing, inherently limited the applicability of randomization in our experimental design.

Blinding	Blinding does not apply for our analysis; The nature of our study, focusing on the comprehensive analysis of immune cell transcriptomes and the diversity of alternative splicing, inherently limited the applicability of blinding in our experimental design.

Reporting for specific materials, systems and methods

We require information from authors about some types of materials, experimental systems and methods used in many studies. Here, indicate whether each material, system or method listed is relevant to your study. If you are not sure if a list item applies to your research, read the appropriate section before selecting a response.

Materials & experimental systems

n/a	Involved in the study	
☐	☒	Antibodies
☐	☒	Eukaryotic cell lines
☒	☐	Palaeontology and archaeology
☒	☐	Animals and other organisms
☒	☐	Clinical data
☒	☐	Dual use research of concern
☒	☐	Plants

Methods

n/a	Involved in the study	
☒	☐	ChIP-seq
☒	☐	Flow cytometry
☒	☐	MRI-based neuroimaging

Antibodies

Antibodies used	anti-CD25, anti-CXCR5, anti-CD3, anti-CCR6, anti-CD45RA, anti-CXCR3, anti-CD4, anti-CCR7, anti-CCR10, anti-CD8, anti-CCR4, anti-CD14, anti-HLA-DR, anti-CD16, anti-CD3, anti-CD123, anti-CD56, anti-CD11c, anti-CD19, anti-CD141, anti-CD15, anti-CD1c, anti-CD19, anti-CD27, anti-CD38, anti-CD3, anti-CD45RA, anti-CD4, anti-IgD, anti-CD8, anti-CCR7
Validation	We conducted a meticulous review process. For each antibody, we followed a multi-step validation protocol which includes, but is not limited to, assessing manufacturer's data, examining relevant literature, and integrating our experimental results.

Eukaryotic cell lines

Policy information about cell lines and Sex and Gender in Research

Cell line source(s)	Cell line sources include LCL (Lymphoblastoid Cell Line) obtained from NA12878 (Coriell Institute) and THP-1 (human monocytic cell line) acquired from ATCC, American Type Culture Collection. All cell lines used are listed in the manuscript with their respective sources.
Authentication	No cell line authentication was performed on these cell lines.
Mycoplasma contamination	The cell line were not tested for contamination.
Commonly misidentified lines (See ICLAC register)	No misidentified cell lines were used in this study.

Plants

Seed stocks	N/A
Novel plant genotypes	N/A
Authentication	N/A
