# Supplementary material for: Long-read sequencing for 29 immune cell subsets reveals disease-linked isoforms
Source: Nat Commun. 2024 May 28;15:4285. doi: 10.1038/s41467-024-48615-4 (PMC11133395; doi:10.1038/s41467-024-48615-4)
Supplement: Supplementary file 1 — Supplementary Information [file 41467_2024_48615_MOESM1_ESM.pdf]

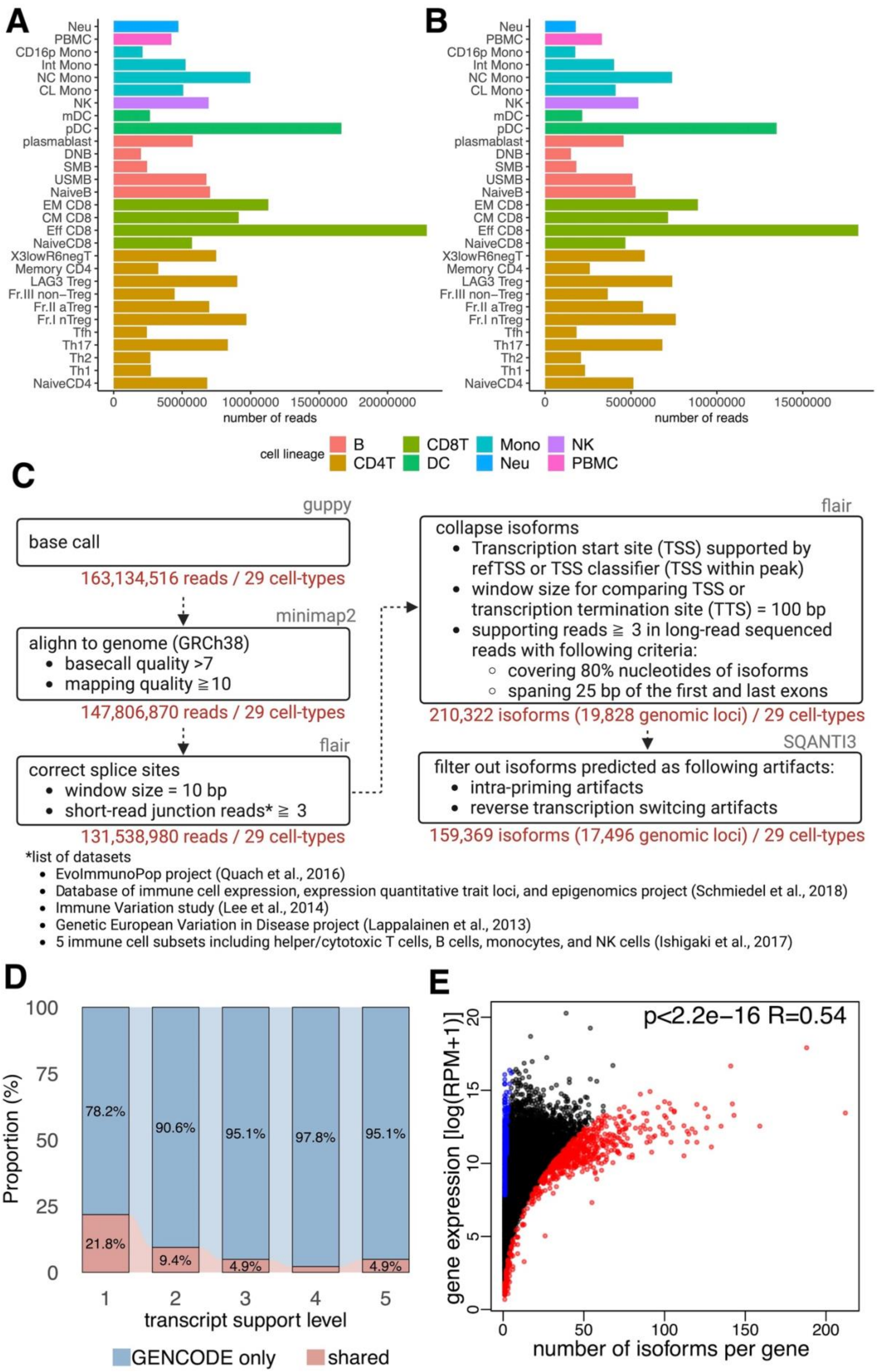
A
B
Neu
PBMC
CD16p Mono
Int Mono
NC Mono
CL Mono
NK
mDC
pDC
plasmablast
DNB
SMB
USMB
NaiveB
EM CD8
CM CD8
Eff CD8
NaiveCD8
X3lowR6negT
Memory CD4
LAG3 Treg
Fr.III non−Treg
Fr.II aTreg
Fr.I nTreg
Tfh
Th17
Th2
Th1
NaiveCD4
0 5000000 10000000 15000000 20000000
number of reads
0 5000000 10000000 15000000
number of reads
cell lineage
B
CD4T
CD8T
DC
Mono
Neu
NK
PBMC
C
guppy
base call
163,134,516 reads / 29 cell-types
minimap2
alighn to genome (GRCh38)
• basecall quality >7
• mapping quality ≧10
147,806,870 reads / 29 cell-types
flair
correct splice sites
• window size = 10 bp
• short-read junction reads* ≧ 3
131,538,980 reads / 29 cell-types
flair
collapse isoforms
• Transcription start site (TSS) supported by refTSS or TSS classifier (TSS within peak)
• window size for comparing TSS or transcription termination site (TTS) = 100 bp
• supporting reads ≧ 3 in long-read sequenced reads with following criteria:
○ covering 80% nucleotides of isoforms
○ spaning 25 bp of the first and last exons
210,322 isoforms (19,828 genomic loci) / 29 cell-types
SQANTI3
filter out isoforms predicted as following artifacts:
• intra-priming artifacts
• reverse transcription switching artifacts
159,369 isoforms (17,496 genomic loci) / 29 cell-types
*list of datasets
• EvoImmunoPop project (Quach et al., 2016)
• Database of immune cell expression, expression quantitative trait loci, and epigenomics project (Schmiedel et al., 2018)
• Immune Variation study (Lee et al., 2014)
• Genetic European Variation in Disease project (Lappalainen et al., 2013)
• 5 immune cell subsets including helper/cytotoxic T cells, B cells, monocytes, and NK cells (Ishigaki et al., 2017)
D
Proportion (%)
100
75
50
25
0
78.2%
21.8%
90.6%
9.4%
95.1%
4.9%
97.8%
95.1%
4.9%
1 2 3 4 5
transcript support level
GENCODE only
shared
E
p<2.2e−16 R=0.54
gene expression [log(RPM+1)]
0 5 10 15 20
0 50 100 150 200
number of isoforms per gene

Supplementary Figure 1. Workflow for generating TRAILS.

(A) Raw sequenced reads in each cell subset.

(B) Mapped sequenced reads on the genome in each cell subset after filtering out reads with a mapping quality (MAPQ) of 10 or less.

(C) Workflow for processing long-read sequenced reads to generate the TRAILS. The gray letters in the upper right corner of the box indicate the name of the tool for each process. The red letters in the lower right corner indicate the number of reads or isoforms that passed the process.

(D) The proportion of isoforms that are shared between the TRAILS and GENCODE (red) and registered only in GENCODE (blue) according to transcript support levels. In GENCODE, isoforms are scored according to how well mRNA and EST alignments match over its full length as follows: 1, all splice junctions of the transcript are supported by at least one non-suspect mRNA; 2, the best supporting mRNA is flagged as suspect or the support is from multiple ESTs; 3, the only support is from a single EST; 4, the best supporting EST is flagged as suspect; 5, no single transcript supports the model structure.

(E) Scatter plot of the number of alternatively spliced isoforms per gene and related gene expression. The red and blue dots indicate genes with the top 5% and bottom 5% of the number of alternatively spliced of isoforms after correction by expression levels of related genes, respectively. The expression value was normalized by reads per million (RPM).

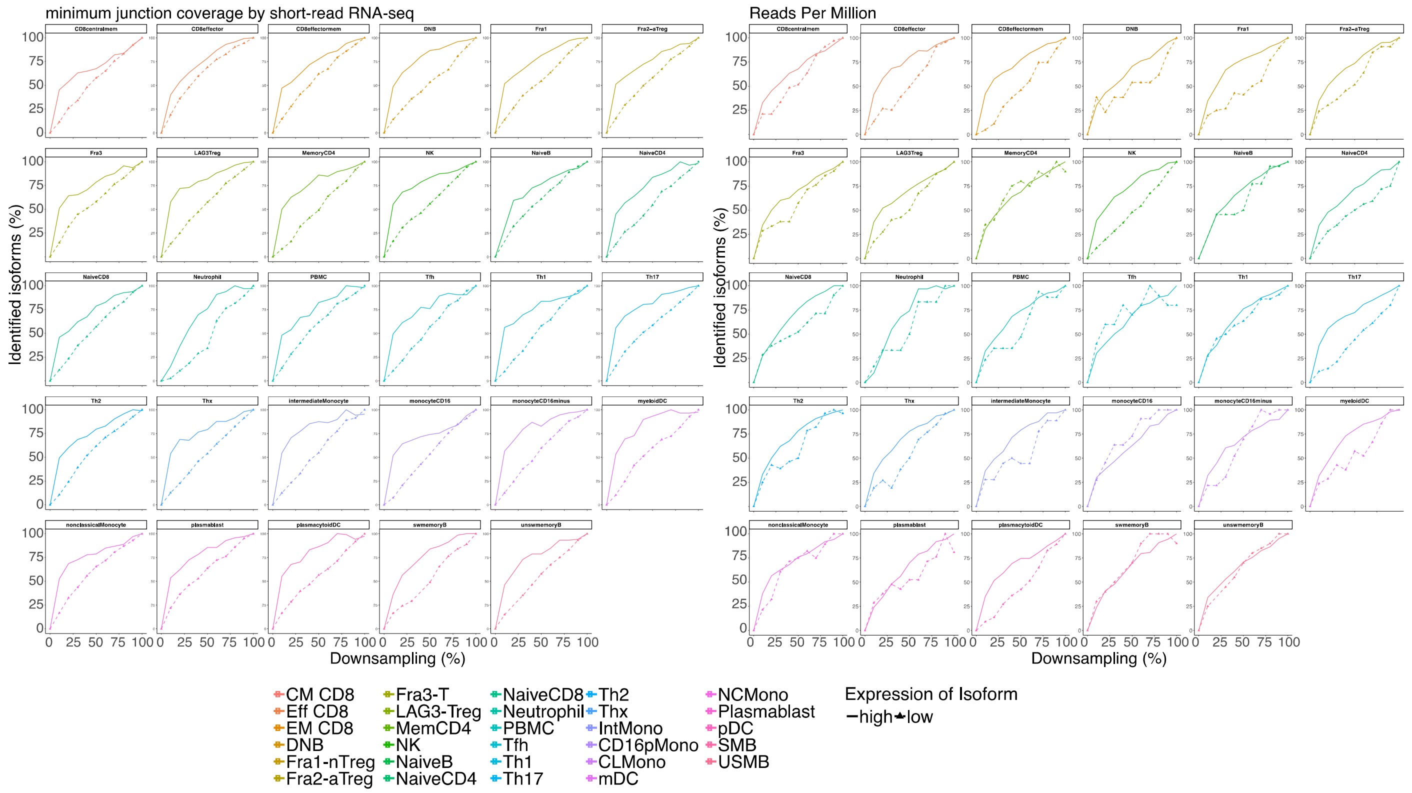

Supplementary Figure 2. Power analysis by downsampling reads. X-axis represents the proportion of downsampling reads. Y-axis represents the proportion of the number of identified isoforms compared with using all reads. The line type represents high-expressed (RPM ≧ 2 in long-read RNA sequencing data [right] and top 25%ile of minimum junction coverage of short-reads used for junction correction [left], solid) and low-expressed (dashed) isoforms in each cell type, respectively.

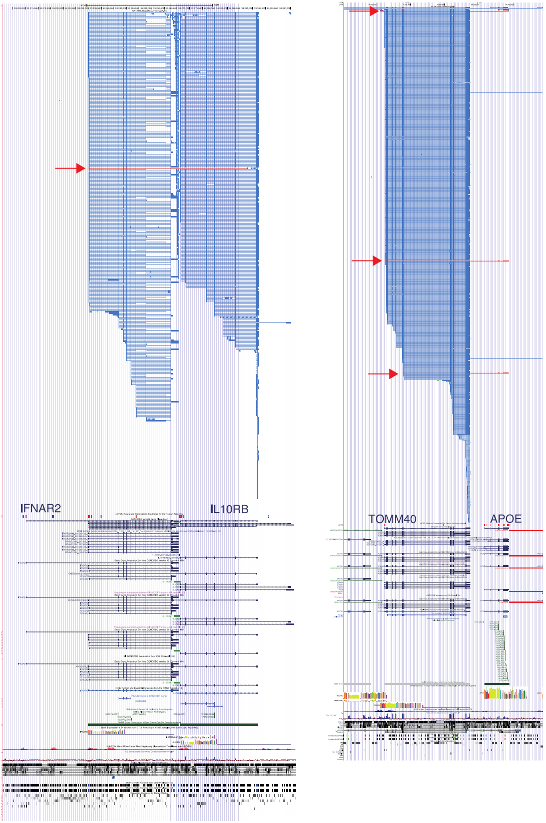

Supplementary Figure 3. Validation of read-through isoforms using direct RNA sequencing. Validated read-through isoforms transcribed from *IFNAR2-IL10RB* region (left) and *TOMM40-APOE* region (right) in lymphoblastoid cell line (GM12878) using ONT direct RNA sequencing. The read-through isoforms are highlighted by red.

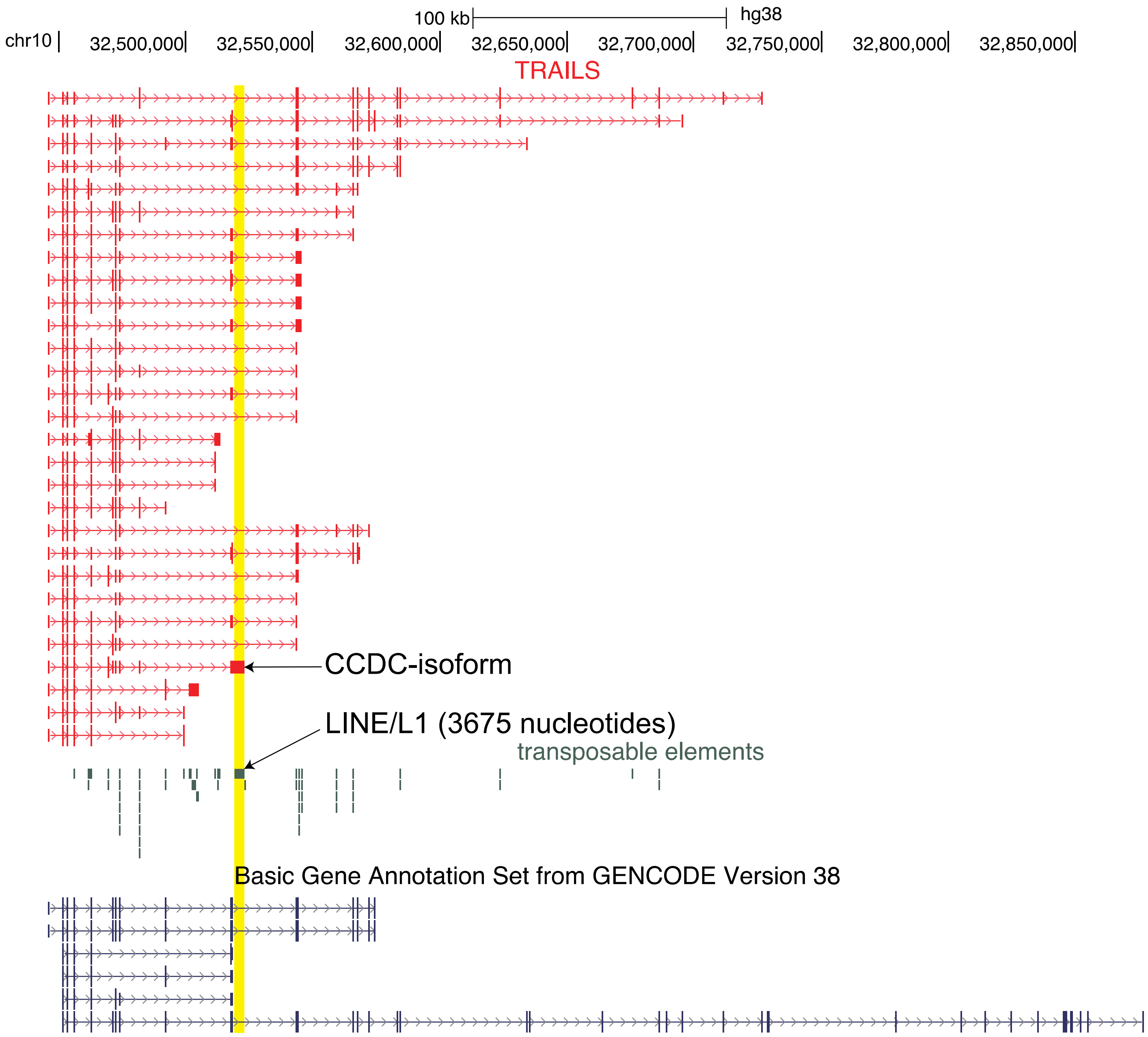

Supplementary Figure 4. Example of isoform inserted transposable elements. These isoforms were transcribed from the *CCDC7* locus, and a particular isoform had an inserted *LINE/L1* highlighted in yellow in the 3′-UTR. The inserted 3′-UTR was not registered in GENCODE, as shown at the bottom.

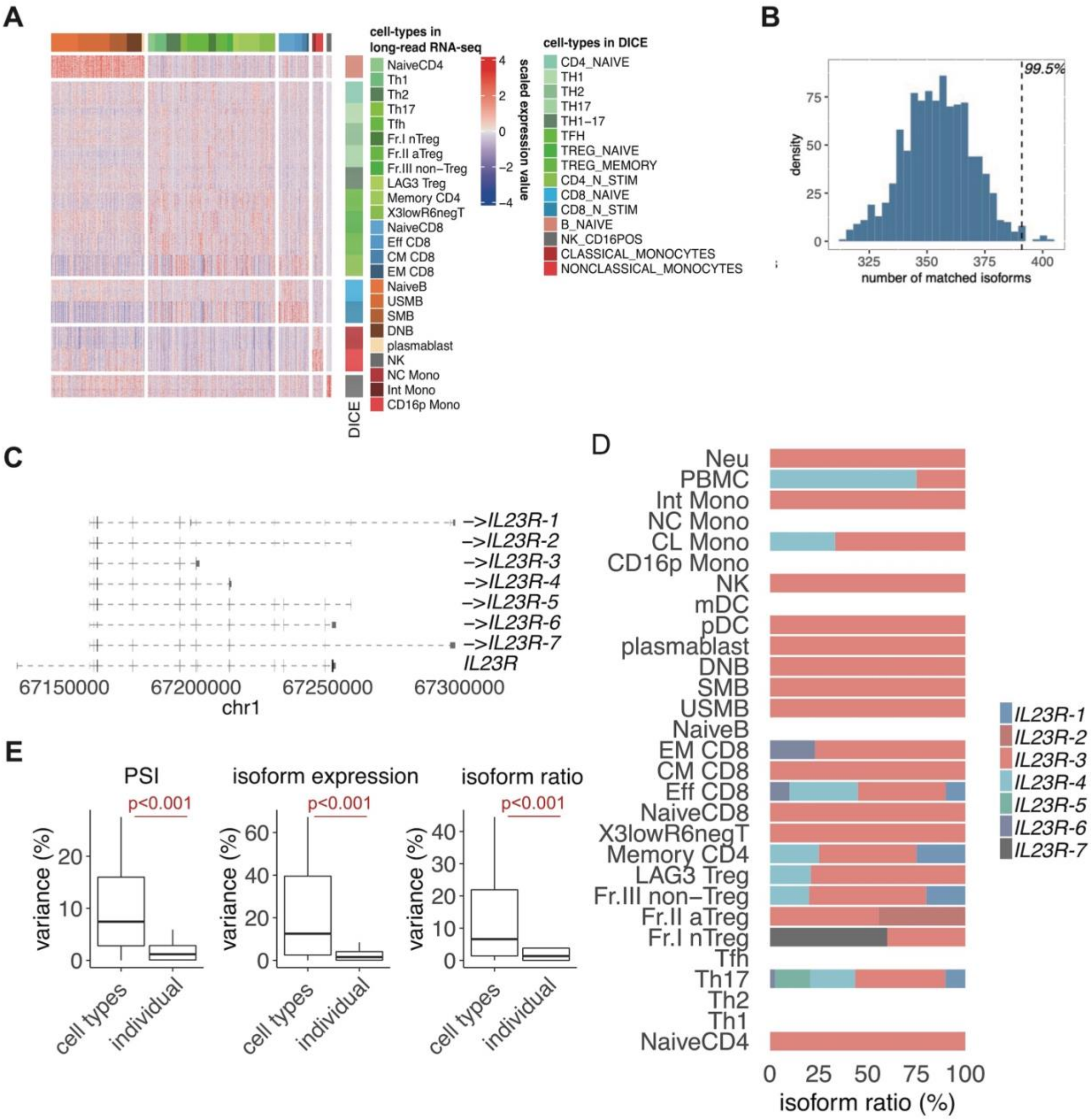

Supplementary Figure 5. The concordant expression of cell-type-specific isoforms in relevant cell types of short-read RNA-seq datasets.

(A) Expression of cell-type-specific isoforms (**Figure 4B**) in cell subsets obtained from short-read RNA-seq datasets as validation. Columns indicate isoforms, and the colored bar at the top indicates the cell type that is specifically expressed in the long-read dataset. Rows indicate cell types in the short-read RNA-seq dataset. Column-wise Z scores of normalized counts are plotted. Cell types are arranged by hierarchical clustering.

(B) Histogram of the number of matched counts between the labels of specifically expressed cell types in the long-read sequencing dataset and the label of the highest expressed cell type in the short-read sequencing dataset by the 1,000 permutation tests. The vertical line shows the true counts of matched isoforms (n=395) between them (permutation p-value = 0.005).

(C) Example of the specifically expressed isoform transcribed from *IL23R* locus. The collapsed gene structure registered in GENCODE is shown at the bottom. The arrow indicates the direction of transcription in the genome.

(D) Isoform ratio in *IL23R*.

(E) Proportion of variance of percent-spliced-in (PSI), isoform expression, and isoform ratio explained by differences in cell types and individuals.

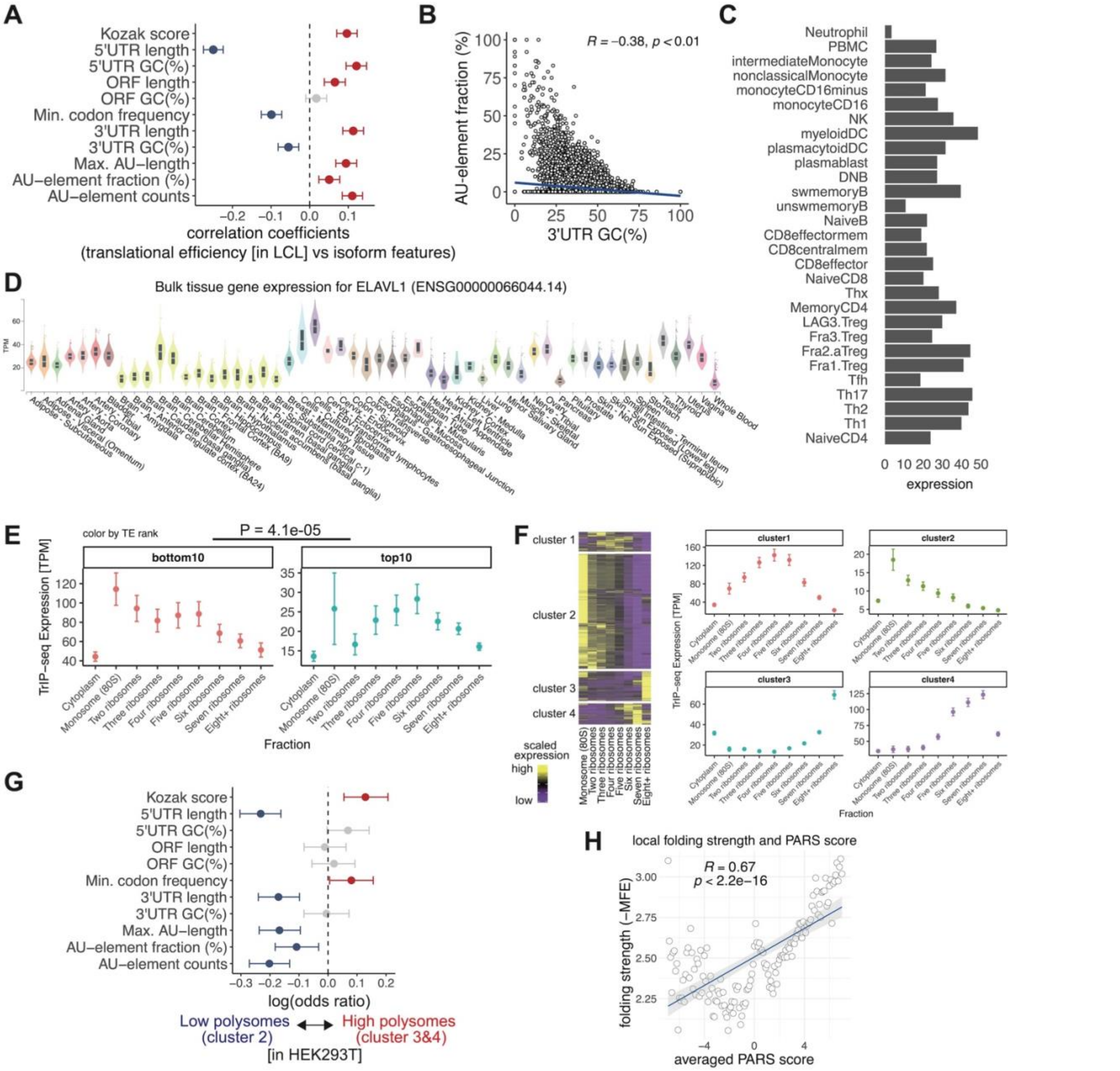

Supplementary Figure 6. Effect of transcript features on translational efficiency.
(A) Meta-transcript distributions of correlation coefficients for transcript features evaluated for translational efficiency in lymphoblastoid cell lines (LCL). Positive coefficients represent positive association for translational efficiency and error bars are bootstrapped 95% confidence intervals. All correlation tests are conducted by two-sided Spearman’s correlation test.
(B) Correlation between 3’-UTR GC contents and AU-element fraction.
(C) Expression of *HuR (ELAVL1)* across 29 immune cell types in TRAILS.
(D) Expression of *HuR (ELAVL1)* across human tissue in GTEx.
(E) Meta-transcript distributions of polysome profiles in HEK293T for isoforms (isoforms in the top 10%ile are more efficiently translated in LCL) by remapping TrIP-seq data obtained. P-value was obtained by comparing two different distributions by Kolmogorov-Smirnov test.
(F) Meta-transcript polysome distributions for four isoform clusters by hierarchical clustering using isoform polysome profiles.
(G) Meta-transcript distributions of odds ratio comparing isoforms with high polysomes (cluster 3 and 4 in panel F) and those with low polysomes (cluster 2) in HEK293T for transcript features.
(H) Correlation between Parallel Analysis of RNA structure (PARS) scores and predicted local folding strength of isoforms.

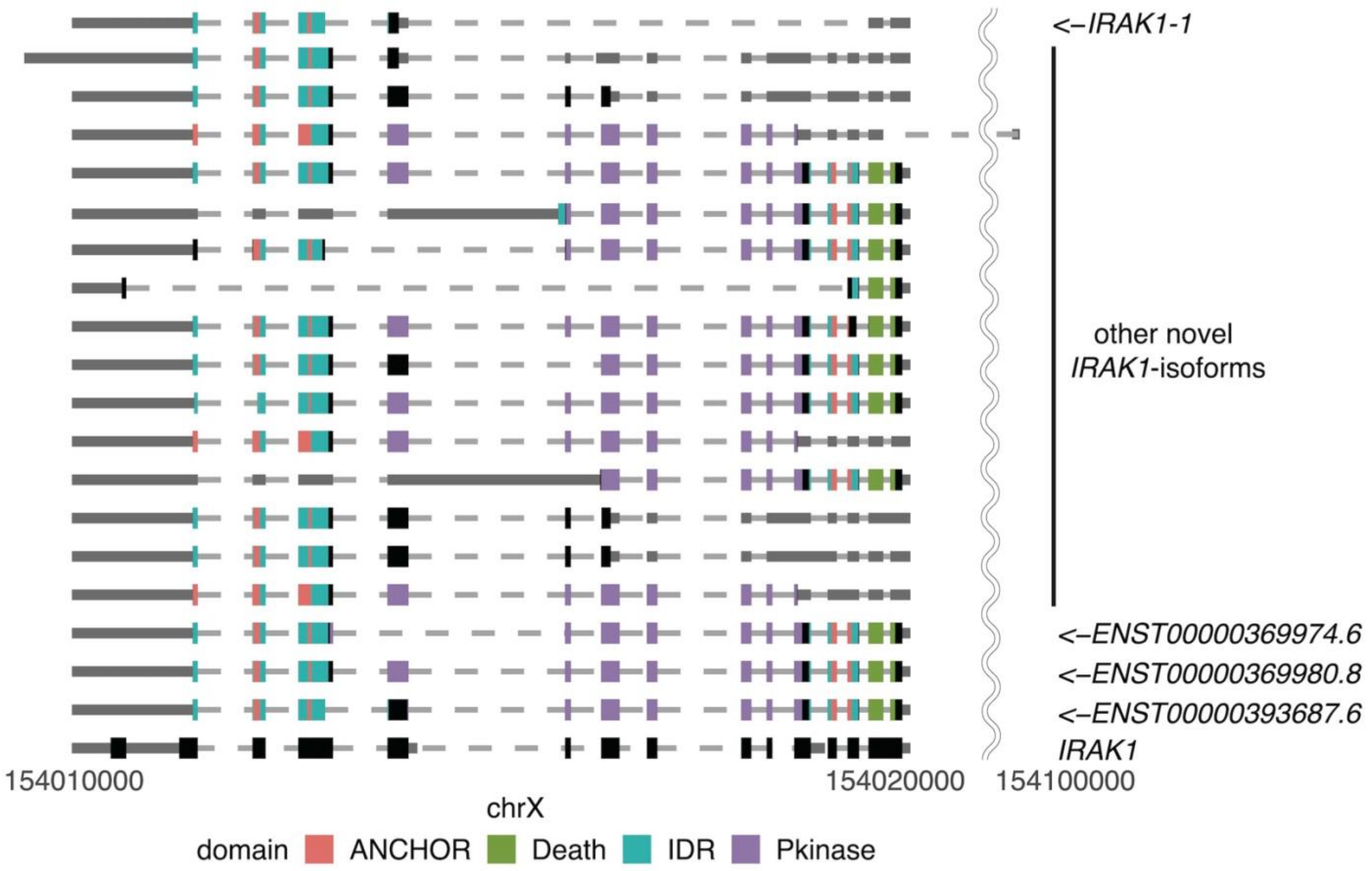

Supplementary Figure 7. The structure of all isoforms that are transcribed from the *IRAK1* locus. The collapsed gene structure registered in GENCODE is shown at the bottom. The arrow indicates the direction of transcription in the genome.

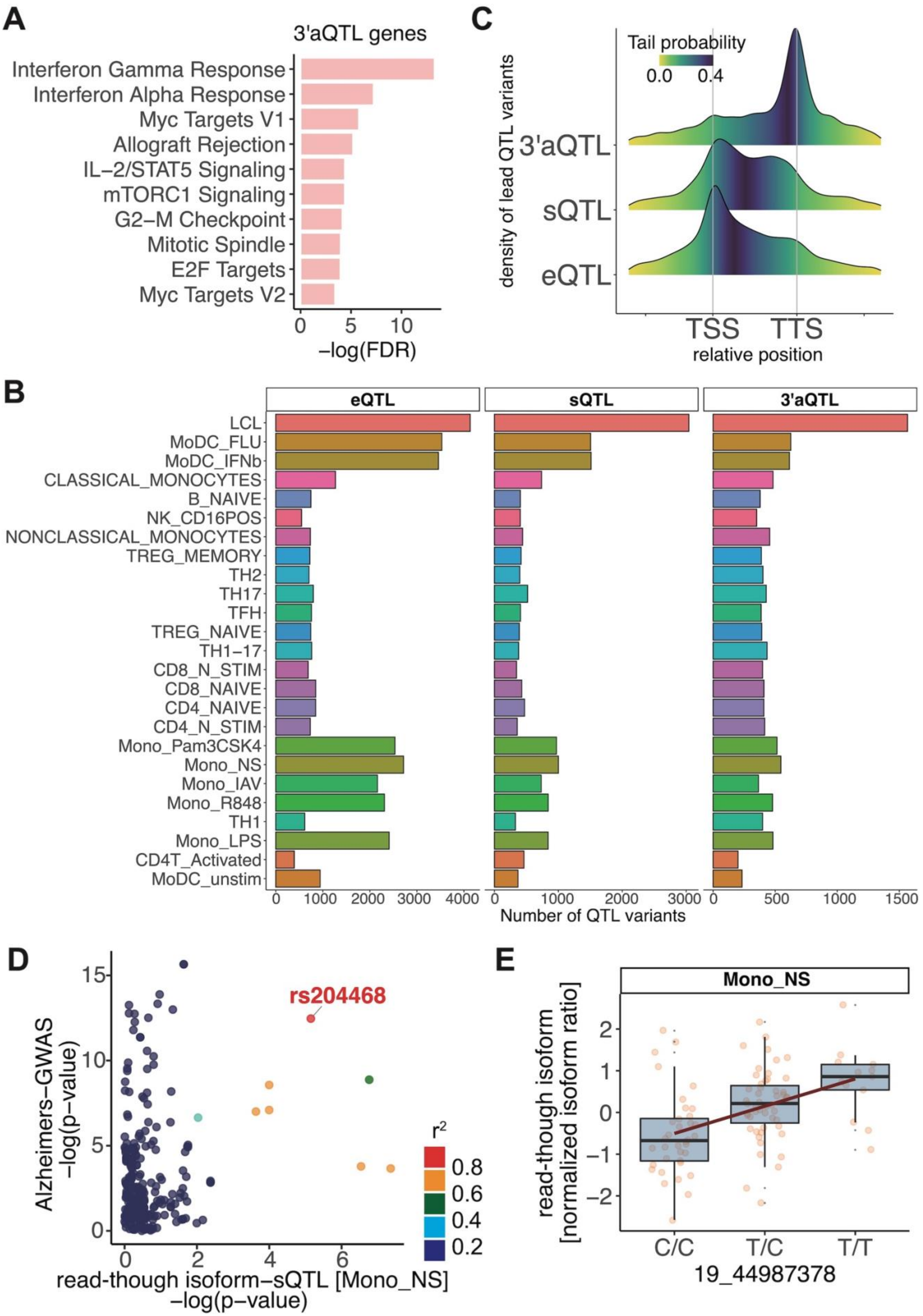
A
3'aQTL genes
Interferon Gamma Response
Interferon Alpha Response
Myc Targets V1
Allograft Rejection
IL−2/STAT5 Signaling
mTORC1 Signaling
G2−M Checkpoint
Mitotic Spindle
E2F Targets
Myc Targets V2
0
5
10
−log(FDR)
C
Tail probability
0.0
0.4
density of lead QTL variants
3'aQTL
sQTL
eQTL
TSS
TTS
relative position
B
eQTL
sQTL
3'aQTL
LCL
MoDC_FLU
MoDC_IFNb
CLASSICAL_MONOCYTES
B_NAIVE
NK_CD16POS
NONCLASSICAL_MONOCYTES
TREG_MEMORY
TH2
TH17
TFH
TREG_NAIVE
TH1−17
CD8_N_STIM
CD8_NAIVE
CD4_NAIVE
CD4_N_STIM
Mono_Pam3CSK4
Mono_NS
Mono_IAV
Mono_R848
TH1
Mono_LPS
CD4T_Activated
MoDC_unstim
0 1000 2000 3000 4000
0 1000 2000 3000
0 500 1000 1500
Number of QTL variants
D
rs204468
Alzheimers−GWAS
−log(p−value)
read−though isoform−sQTL [Mono_NS]
−log(p−value)
r^2
0.8
0.6
0.4
0.2
E
Mono_NS
read−though isoform
[normalized isoform ratio]
C/C
T/C
T/T
19_44987378

Supplementary Figure 8. Characteristics of QTL genes and QTL variants.

(A) Characteristics of alternative polyadenylation (APA) quantitative trait loci (3′aQTL)-genes. Enriched pathways in 3′aQTL-genes

(B) The number of total lead QTL variants (nominally significant p-values < 1×10^{-5}) for each eGene (eQTL), junction (sQTL), and isoform (3’aQTL), sorted by the sample sizes.

(C) The distribution of lead QTL variants in the relative position.

(D) Colocalization plot of trQTL of the read-through isoform transcribed from the *TOMM40_APOE* locus and GWAS of Alzheimer's disease in non-stimulated monocytes.

(E) QTL plot of normalized isoform ratio of the read-through isoform transcribed from the *TOMM40_APOE* locus in non-stimulated according to each genotype of rs204468.

Abbreviations of cell-types in short-read RNA-seq datasets and their related cohorts were as follows: LCL, lymphoblastoid cell-lines [GEUVADIS]; Mono_NS, monocytes without treatment [EvoImmunoPop]; Mono_LPS, monocytes treated with TLR4 agonist [EvoImmunoPop]; Mono_Pam3CSK4, monocytes treated with TLR 1/2 agonist [EvoImmunoPop]; Mono_R848, monocytes treated with TLR 7/8 agonist [EvoImmunoPop]; Mono_IAV, monocytes treated with H1N1 Influenza A virus [EvoImmunoPop]; CD4T_Activated, $CD4^+$ T cells treated with anti-CD3/CD28 antibody [ImmVar]; MoDC_unstim, monocyte-derived dendric cells without treatment [ImmVar]; MoDC_IFNb, monocyte-derived dendric cells treated with IFNb [ImmVar];

MoDC_FLU, monocyte-derived dendric cells treated with H1N1 Influenza A virus [ImmVar]; CD4_NAIVE, naïve $CD4^+$ T cells [DICE]; CD4_N_STIM, naïve $CD4^+$ T cells treated with anti-CD3/CD28 antibody [DICE]; TH1, T_H1 cells [DICE]; TH2, T_H2 cells [DICE]; TH1-17, T_H1-17 cells [DICE]; TH17, T_H17 cells [DICE]; TFH, T_{FH} cells [DICE]; TREG_NAIVE, naïve T_{REG} cells [DICE]; TREG_MEMORY, memory T_{REG} cells [DICE]; CD8_NAIVE, naïve $CD8^+$ T cells [DICE]; CD8_N_STIM, Naïve $CD8^+$ T cells treated with anti-CD3/CD28 antibody [DICE]; B_NAIVE, naïve B cells [DICE]; CLASSICAL_MONOCYTES, classical monocytes [DICE]; NONCLASSICAL_MONOCYTES, nonclassical monocytes [DICE]; NK_CD16POS, NK cells [DICE].

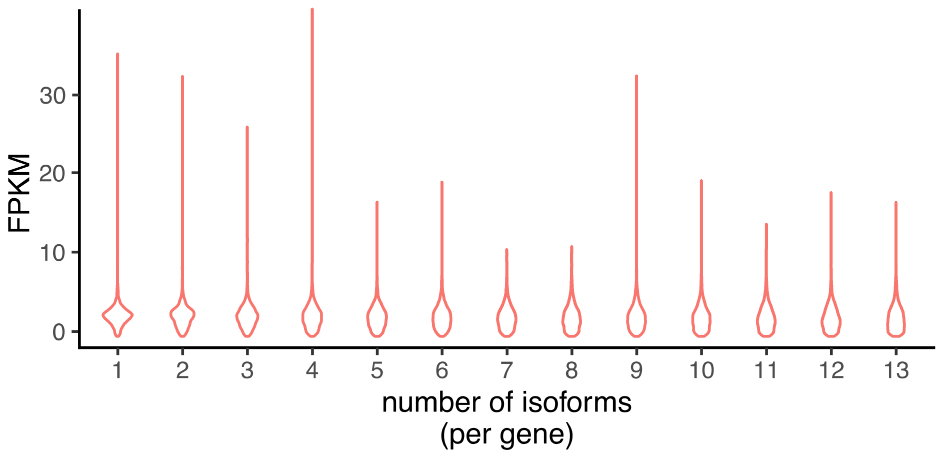

Supplementary Figure 9. Performance of mapping to TRAILS using a simulated paired-end short-read RNA sequencing dataset (equal FPKM for all isoforms).

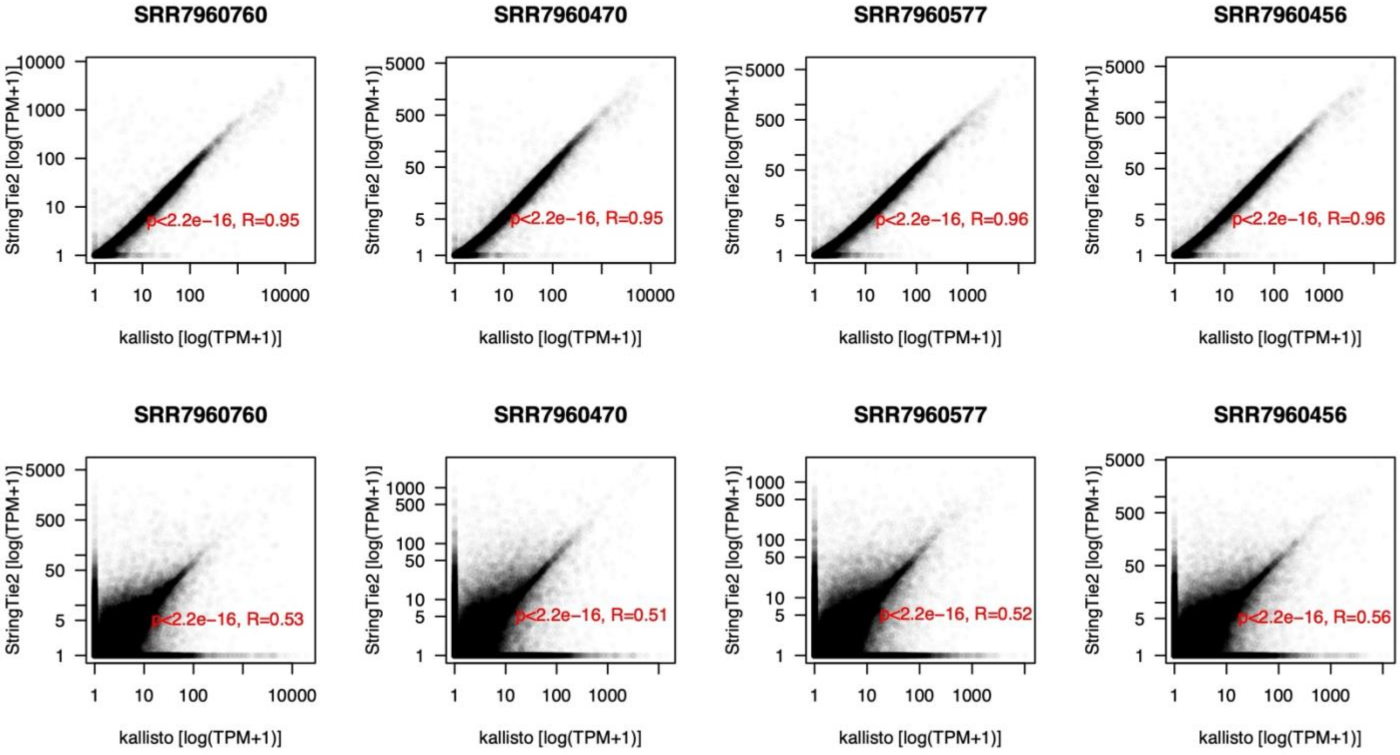

Supplementary Figure 10. Comparison of quantification tools. The top four and bottom four scatter plots were drawn using expression values at the gene levels and isoform level, respectively. Each dot represented genes (top) and isoforms (bottom). Four samples were randomly selected from short-read RNA-seq datasets of DICE. The boxes contain Spearman's correlation coefficient (R) and p-value.
